# Influenza‐associated severe acute respiratory infections in 2 sentinel sites in Lebanon—September 2015 to August 2016

**DOI:** 10.1111/irv.12527

**Published:** 2018-03-09

**Authors:** Majd Saleh, Lara Bazzi, Ebstissam Ismail, Lina Mroueh, Nisrine Jammal, Amgad Elkholy, Pamela Mrad, Ahmad Al Samadi, Ahmad Hijazi, Firass Abiad, Ghazi Nsouli, Alissar Rady, Wasiq Khan, Mamunur Malik, Pierre Zalloua, Walid Ammar, Nada Ghosn

**Affiliations:** ^1^ Epidemiological Surveillance Program Lebanese Ministry of Public Health Beirut Lebanon; ^2^ Saida Governmental University Hospital Saida Lebanon; ^3^ Rafik Hariri University Hospital Beirut Lebanon; ^4^ National Influenza Laboratory Rafik Hariri University Hospital Beirut Lebanon; ^5^ World Health Organization Eastern Mediterranean Regional Office Cairo Egypt; ^6^ World Health Organization Lebanon Country Office Beirut Lebanon; ^7^ Lebanese American University Beirut Lebanon

**Keywords:** Eastern Mediterranean Region, influenza, Lebanon, sentinel surveillance, severe acute respiratory infections

## Abstract

**Background:**

Given the sparse information on the burden of influenza in Lebanon, the Ministry of Public Health established a sentinel surveillance for severe acute respiratory infections (SARI) to identify the attribution of influenza to reported cases. We aim to highlight the proportion of influenza‐associated SARI from September 1st, 2015 to August 31st, 2016 in 2 Lebanese hospitals.

**Methods:**

The study was conducted in 2 sentinel sites located in Beirut suburbs and southern province of Lebanon. WHO's 2011 standardized SARI case definition was used. Data from September 1, 2015 to August 31, 2016 were reviewed, and all‐cause hospital admission numbers were obtained. Nasopharyngeal swabs were collected and tested by RT‐PCR. Descriptive and bivariate analyses were conducted using STATA 13.

**Results:**

The 2 sentinel sites reported 746 SARI cases during the studied time frame: 467 from the southern province site and 279 from the Beirut suburbs site. SARI reports peaked between January and March 2016. All, except 4, cases were sampled, and a co‐dominance of influenza B (43%) and influenza A (H1N1) (41%) was evident. A high proportion of cases was reported in children <2 years 274 (37%). The proportional contribution of influenza‐associated SARI to all‐cause hospital admissions was high in children <2 years in the south (4.5% [95% CI: 3.1‐6.5]) and in children <5 years in Beirut (0.7% [95% CI: 0.6‐0.8]).

**Conclusion:**

This is the first study to highlight the proportion of influenza‐associated SARI in 2 hospitals in Lebanon. The findings will be beneficial for supporting respiratory prevention and immunization program policies.

## 1 INTRODUCTION

Following the 2009 H1N1 pandemic, the importance of having appropriate surveillance systems to monitor influenza trends became evident globally.^1^ However, there remains scarce information about the burden of influenza in the Eastern Mediterranean Region (EMR).^2^

Lebanon is divided into 6 governorates, constituting a wide range of private and public hospitals.^3^ In the past, the Ministry of Public Health (MOPH) relied on several sources to identify acute respiratory infections such as intensive care unit‐based surveillance, school absenteeism system, and admissions covered by the MOPH health insurance data. All these programs relied solely on passive surveillance, and most often had no laboratory confirmation. To fill this knowledge gap and understand better the circulation of influenza viruses in Lebanon, the World Health Organization (WHO) recommended the establishment of a severe acute respiratory infections (SARI) sentinel surveillance, under its “Pandemic Influenza Preparedness Framework” (PIP).^4^ The Epidemiologic Surveillance Unit (ESU) at the MOPH initiated the SARI sentinel surveillance system in December 2014 in collaboration with the National Influenza Center (NIC) located at Rafik Hariri University Hospital in Lebanon.

In this study, we aim to highlight the results of estimating the proportion of influenza‐associated SARI from September 1st, 2015 to August 31st, 2016 in 2 sentinel sites located in Beirut suburbs and southern governorate of Lebanon for evidence‐based interventions and control programs such as immunization and awareness campaigns.

## 2 METHODS

### 2.1 SARI surveillance

Data on SARI cases were collected in 2 pilot sentinel sites that are public general hospitals covering Beirut suburbs and the southern province of Lebanon. At the start of this pilot, a total of 11 general hospitals were selected for the SARI surveillance. However, this study will present data for the 2 pilot sites as they had complete seasonal data.

A focal point appointed by the sentinel site was responsible for case finding and reporting since February 2015. The SARI case definition was as per WHO guidelines^1^: any patient having an acute respiratory infection with (i) fever or history of fever ≥38°C); (ii) cough; (iii) illness within 10 days; and (iv) requiring hospitalization.

The focal point collected data through passive and active surveillance: passively by receiving notification of cases from medical teams within the hospital and actively by visiting all hospital wards searching for cases. The same method of case finding was applied at both sites.

Specimens were collected using nasopharyngeal or oropharyngeal swabs and stored in viral transport media (VTM) at the sentinel site laboratory for 48‐72 hours at 2‐4°C. If the storage time exceeded 72 hours, the specimens were stored at −80°C. The specimens were transferred to the National Influenza Center (NIC) for testing in a cold box with frozen ice packs. At the NIC, specimens were tested by RT‐PCR for influenza types A and B, and type A was further subtyped. Influenza virus RNA was extracted from 200 mL of VTM using High Pure Viral Nucleic Acid Kit (Roche, Mannheim, Germany). RT‐PCR was conducted on 5 mL of extracted RNA using the Superscript III Platinum One‐step qRT‐PCR system kit, where the sample was mixed with influenza A primers (InfA, A/H1pmd09, A/pmd infA, A/H3, A/H5a, A/H5b, A/H7), influenza B primers (InfB, Yamagata, and Victoria lineages), and RP primers. The specimen is run on Bio‐Rad IQ5 thermal cycler (Bio‐Rad, Hercules, CA, USA) with the following conditions: 50°C for 30 minutes, 95°C for 2 minutes, 95°C for 15 seconds, run for 45 cycles, and 55°C for 30 seconds.

Data on demographic, clinical, and laboratory findings were entered into a SARI database (EpiData 3.1; The EpiData Association, Odense, Denmark) and sent via email to the Epidemiologic Surveillance Unit (ESU) at the MOPH on a weekly basis.

### 2.2 Influenza‐associated SARI study

To ensure that a complete influenza season is demonstrated, SARI data from September 1st, 2015 to August 31st, 2016 were used for the analysis.

Data reported from September 1st, 2015 to August 31st, 2016 were reviewed at the sentinel site, and total numbers of all‐cause hospital admissions were obtained. An analysis plan was developed based on WHO's guideline “A Manual for Estimating Disease Burden Associate with Seasonal Influenza,” and analysis was conducted accordingly.^5^ Descriptive analysis was carried out for categorical data and shown as frequencies and proportions. Bivariate analysis for comparing groups was conducted using Pearson's chi‐square test. The analysis was carried out using STATA 13 (StataCorp LLC, College Station, TX, USA) and Microsoft Excel 2007.

## 3 RESULTS

The total number of reported cases from the 2 sentinel sites was 746, of which 467 (63%) were from the southern sites and 279 (37%) from the Beirut suburbs site. At the southern site, children <2 years of age constituted 208 (45%) of all reported cases and 275 (59%) of all cases were males. Only 4 (1%) cases were admitted to the intensive care unit and required ventilation. Majority of cases resided in the southern governorate of Lebanon, with a total of 437 (94%) cases. Similarly, data from the Beirut suburb site revealed 66 (23%) of cases were children <2 years of age and 143 (51%) were males. The reported cases requiring intensive care admissions were 77 (28%) and 28 (10%) required ventilation. Cases admitted to the Beirut suburbs hospital were predominantly residents of the capital Beirut and the district of Baabda, with a total of 85 (30%) cases each.

The highest wave of SARI reports for both the southern and Beirut suburbs sentinel sites was evident between weeks 1 and 10 of the year 2016 (January 4 to March 13).

**Figure 1:**
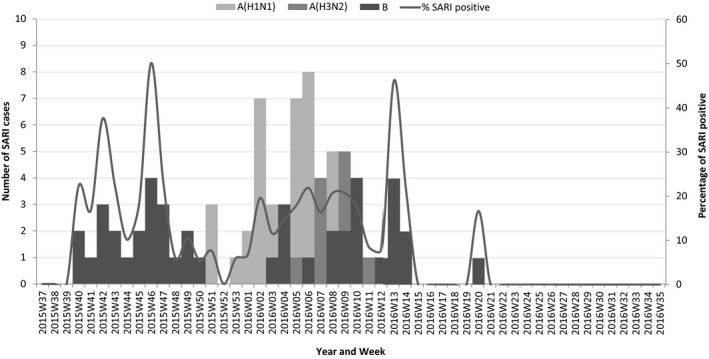
Influenza virus detection by (sub)type and percent positive

Samples for influenza testing were collected from all SARI cases 279 (100%) at the Beirut suburb site and from 463 (99%) at the southern site, of which 30 (11%) and 70 (15%) were positive, respectively. At the southern site, influenza B accounted for 29 (41%) of positive cases followed by 27 (39%) influenza A (H1N1)pdm09 and 14 (20%) influenza A (H3N2). Of the 30 cases positive for influenza B, children <2 years of age accounted for 67%. On the other hand, at the Beirut suburbs sentinel site, influenza B and influenza A (H1N1)pdm09 had the same numbers of 14 (47%), while influenza A (H3N2) constituted 2 (6%) of positives at the site (Figure 1).

Looking at results of the proportion of influenza positive by age groups, the 2 sentinel sites had significantly different distributions (*P* < .05). At the southern sentinel site, ages 15‐49 years sampled for influenza testing had the highest positive results with a proportion of influenza of positive 26%, followed by ages 65 years and older with a proportion of influenza positive of 22%. At the Beirut suburb sites, ages 5‐15 years had the highest proportion of influenza positive (21%) followed by ages 50‐64 years (15%; Table 1).

**Table 1:** Proportion of influenza‐associated severe acute respiratory infections (SARI) and influenza‐associated SARI to all hospital admissions from September 1st, 2015 to August 31st, 2016

Sentinel sites	Indicator	Age group					
		<2	2‐4	5‐14	15‐49	50‐64	≤65
South	Total SARI reported	208	108	69	50	14	18
	Total SARI collected for Laboratory tests	207	106	68	50	14	18
	Total SARI positive	29	11	11	13	2	4
	Proportion of influenza positive (%)	14	10	16	26	20	22
	SARI positive for A(H1N1)pdm09	7	7	1	9	1	2
	SARI positive for A(H3N2)	2	2	6	2	1	1
	SARI positive for influenza B	20	2	4	2	0	1
	Total site all‐cause admissions	647	725	721	3212	1215	1449
	Proportional contribution of influenza‐associated SARI to all site admissions (%) [95% CI]	4.5 [3.1‐6.5]	1.5 [.8‐2.7]	1.5 [.8‐2.7]	0.4 [.2‐.7]	0.2 [.1‐.6]	0.3 [.1‐.8]
Beirut	Total SARI reported	66	33	14	59	46	61
	Total SARI collected for Laboratory tests	66	33	14	59	46	61
	Total SARI positive	2	4	3	7	7	7
	Proportion of influenza positive (%)	3	12	21	12	15	11
	SARI positive for A(H1N1)pdm09	0	1	3	4	3	3
	SARI positive for A(H3N2)	0	0	0	0	0	2
	SARI positive for influenza B	2	3	0	3	4	2
	Total site all‐cause admissions	365	533	1013	5612	3190	2795
	Proportional contribution of influenza‐associated SARI to all site admissions (%) [95% CI]	0.5 [0.1‐2.0]	0.7 [0.3‐1.9]	0.3 [0.1‐0.9]	0.1 [0.05‐0.2]	0.2 [0.1‐0.4]	0.2 [0.1‐0.4]

The overall proportion of influenza‐associated SARI to all‐cause hospital admissions at the southern sentinel site was 0.9% [95% CI: 0.7‐1.1], while the Beirut suburb site was 0.2% [95% CI: 0.1‐0.3]. The proportional contribution of influenza‐associated SARI to all hospital admissions by age group in the southern site showed children <5 years of age had a high proportion (2.9% [95% CI: 2.1‐3.9]), yet with further age breakdown it was higher in children <2 years of age in particular (4.5% [95% CI: 3.1‐6.5]; Table 1). In the Beirut suburbs site, on the other hand, children <5 years of age had a higher proportion (0.7 [95% CI: 0.6‐0.8]) than children <2 years of age (0.5% [95% CI: 0.1‐2]).

## 4 DISCUSSION

Two sentinel sites were piloted for influenza‐associated SARI testing and reported 746 SARI cases during the studied time frame. The peak of SARI reports was between January and March 2016. A co‐dominance of influenza B (43%) and influenza A (H1N1) (41%) was evident. A high proportion of the SARI cases were reported in children <2 years of age (37%). The proportional contribution of influenza‐associated SARI to all‐cause hospital admissions was high in children <2 years of age in the south (4.5% [95% CI: 3.1‐6.5]) and in children <5 years in Beirut (0.7% [95% CI: 0.6‐0.8]).

Severe acute respiratory infections surveillance systems have proved to be a good source for obtaining estimates of influenza trends as well as estimating the proportion of influenza‐related SARI.^6^ We defined the proportional contribution of influenza‐associated SARI to all‐cause hospital admissions from all wards rather than using catchment population; hence, incidence rates were not obtained. Calculating the catchment population in Lebanon is challenging due to its decentralized system; however, this may be overcome by specific catchment estimates in the future.

While reviewing the data at the sentinel sites, the possibility of using ICD‐10 codes in addition to reported SARI cases was explored; however, this was finally disregarded as codes used in WHO's manual did not correspond to what codes were used at the hospital admission logs. ICD‐10 for influenza (J09‐J11) are scarcely used given the late or lack of influenza results by the time of patient discharge. Most codes used are based on upper respiratory tract infections (J03‐J04, J06, J11, J18, J20‐J21) or specific symptoms such as fever (R50) and cough (R05). This made it difficult for estimating the total number of respiratory‐related admissions at the sites.

The findings of the study revealed that the proportion of influenza‐associated SARI to all hospital admissions at the southern sentinel sites was 0.9% [95% CI: 0.7‐1.1], while in Beirut, it was 0.2% [95% CI: 0.1‐0.3]. There are several explanations for the small proportion shown at the 2 sentinel sites. One explanation is that the denominator used was all‐cause admissions and was not narrowed down to respiratory illness due to the aforementioned difficulty of having total numbers of respiratory illness at the sites. Additional studies are suggested to study the hospital profiles and the overall respiratory illness‐related admissions at the sentinel sites.

Taking the southern sentinel site alone, and dividing into age groups, children less than the age of 5 years have a high influenza‐associated SARI to all‐cause hospital admissions (2.9% [95% CI: 2.1‐3.9]). Children <2 years of age have a higher proportion of 4.5% [95% CI: 3.1‐6.5] when compared to the Beirut suburb site (0.5% [95% CI: .1‐2]). An explanation for these slight differences might be the hospital profile and differences in admission patterns in particular. The southern district had higher admissions in pediatric wards (80%) compared to the Beirut suburb site (30%). These proportions among children, however, might still be considered small given other pathogens might be causing increased admissions of children <5 years such as respiratory syncytial virus (RSV) and others 6, 7, 8. Routinely testing for a range of non‐influenza respiratory pathogens to find out what other pathogens might be the cause of SARI admissions is recommended.

Comparing our data with the literature, the influenza‐associated SARI seemed to be low, yet the fact that children less than 5 years of age have the highest risk is commonly reported. Data from 2008 to 2014 in Jordan revealed that 57% of influenza positive SARI cases are children aged less than 5 years old.^9^ In Oman, children less than 5 years had the highest influenza‐associated SARI incidence (32‐42 per 10 0000).^10^ Another study conducted in 3 selected provinces in Iran also revealed the highest risk groups among SARI cases were children less than 5 years of age with an overall influenza‐associated SARI of 29/10 0000 [95% CI: 16.8‐43.8/10 0000].^11^ In Kenya, most SARI cases reported were children less than 5 years of age with influenza‐associated hospitalized SARI for the same age group of 2.7/1000 [95% CI: 1.8‐3.9/1000].^7^ In a burden of influenza study conducted in England from 2000 to 2008, it was shown that the highest influenza admissions are in children <5 years of age as well (1.9/1000 [95% CI ± 0.023/1000]).^8^ The findings in the literature suggest that the results of the SARI surveillance system in Lebanon are comparable with the results in other countries. Children less than the age of 5 years are the most at risk, and as in the southern province site in Lebanon, they do form a burden on the hospital during the cold season in Lebanon. With the further development of sentinel SARI surveillance in Lebanon by including other sentinel sites and determination of the catchment population, we will have better estimates of influenza‐associated SARI in the future.

The limitations of the study are many, and 1 major limitation is that only 2 sentinel sites were selected for estimating influenza‐associated SARI. The sentinel sites were selected given their complete seasonal data, yet this can lead to difficulty generalizing the findings. In addition, a relatively low number of influenza positive cases was observed. Therefore, the data should be interpreted carefully and may not be directly compared to data from the southern province. The other aspect is that SARI surveillance only covers severe cases admitted to the hospital, and hence, the estimates here include only severe influenza cases. A general understanding of influenza burden in Lebanon can be developed once the results in SARI surveillance system are combined with an influenza‐like illnesses (ILI) sentinel surveillance system once initiated.

This is the first study to highlight the proportion of influenza‐associated SARI cases using data from 2 governmental hospitals in Beirut suburbs and the southern governorate in Lebanon and using a national surveillance system. The study is a basis for replication in the other SARI sentinel sites to be able to have national findings on influenza‐associated SARI. This study is the first step in better understanding severe influenza in Lebanon, and this information can be used for better control programs such as immunization and awareness campaigns.
